# Disruption as opportunity: Impacts of an organizational health equity intervention in primary care clinics

**DOI:** 10.1186/s12939-018-0820-2

**Published:** 2018-09-27

**Authors:** Annette J. Browne, Colleen Varcoe, Marilyn Ford-Gilboe, C. Nadine Wathen, Victoria Smye, Beth E. Jackson, Bruce Wallace, Bernadette (Bernie) Pauly, Carol P. Herbert, Josée G. Lavoie, Sabrina T. Wong, Amelie Blanchet Garneau

**Affiliations:** 1 School of Nursing, The University of British Columbia, T201-2211 Wesbrook Mall, Vancouver, BC V6T 2B5 Canada; 2 Arthur Labatt Family School of Nursing, Western University, FIMS & Nursing Building, London, ON N6A 5B9 Canada; 3 Faculty of Information & Media Studies, Western University, FIMS & Nursing Building, London, ON N6A 5B9 Canada; 4 Public Health Agency of Canada, 785 Carling Avenue, AL 6809B, Ottawa, ON K1A 0K9 Canada; 5 School of Social Work, University of Victoria, PO Box 1700, STN CSC, Victoria, BC V8W 2Y2 Canada; 6 Canadian Institute for Substance Use Research, and School of Nursing, University of Victoria, Victoria, BC V8W 2Y2 Canada; 7 School of Population and Public Health, The University of British Columbia, and Centre for Studies in Family Medicine, The Western Centre for Public Health and Family Medicine, Western University, London, ON N6A 3K7 Canada; 8 Department of Community Health Sciences and Ongomiizwin – Research, Indigenous Institute of Health and Healing, Rady Faculty of Health Sciences, University of Manitoba, Winnipeg, MB MB R3E 3P5 Canada; 9 Centre for Health Services and Policy Research and School of Nursing, University of British Columbia, T201-2211 Wesbrook Mall, Vancouver, BC V6T 2B5 Canada; 10 Faculty of Nursing, Universite de Montreal, PO Box 6128, Centre-ville Station, Montreal, QC H3C 3J7 Canada

**Keywords:** Health equity, Health services research, Trauma informed care, Trauma- and violence-informed care, Structural violence, Attitude of health personnel, Harm reduction, Primary care, Vulnerable populations, Indigenous populations, Health care disparities, Health services accessibility

## Abstract

**Background:**

The health care sector has a significant role to play in fostering equity in the context of widening global social and health inequities. The purpose of this paper is to illustrate the process and impacts of implementing an organizational-level health equity intervention aimed at enhancing capacity to provide equity-oriented health care.

**Methods:**

The theoretically-informed and evidence-based intervention known as ‘EQUIP’ included educational components for staff, and the integration of three key dimensions of equity-oriented care: cultural safety, trauma- and violence-informed care, and tailoring to context. The intervention was implemented at four Canadian primary health care clinics committed to serving marginalized populations including people living in poverty, those facing homelessness, and people living with high levels of trauma, including Indigenous peoples, recent immigrants and refugees. A mixed methods design was used to examine the impacts of the intervention on the clinics’ organizational processes and priorities, and on staff.

**Results:**

Engagement with the EQUIP intervention prompted increased awareness and confidence related to equity-oriented health care among staff. Importantly, the EQUIP intervention surfaced tensions that mirrored those in the wider community, including those related to racism, the impacts of violence and trauma, and substance use issues. Surfacing these tensions was disruptive but led to focused organizational strategies, for example: working to address structural and interpersonal racism; improving waiting room environments; and changing organizational policies and practices to support harm reduction. The impact of the intervention was enhanced by involving staff from all job categories, developing narratives about the socio-historical context of the communities and populations served, and feeding data back to the clinics about key health issues in the patient population (e.g., levels of depression, trauma symptoms, and chronic pain). However, in line with critiques of complex interventions, EQUIP may not have been maximally disruptive. Organizational characteristics (e.g., funding and leadership) and characteristics of intervention delivery (e.g., timeframe and who delivered the intervention components) shaped the process and impact.

**Conclusions:**

This analysis suggests that organizations should anticipate and plan for various types of disruptions, while maximizing opportunities for ownership of the intervention by those within the organization. Our findings further suggest that equity-oriented interventions be paced for intense delivery over a relatively short time frame, be evaluated, particularly with data that can be made available on an ongoing basis, and explicitly include a harm reduction lens.

## Background

Recently, the Institute for Health Care Improvement [1] released an urgent call for health care organizations to make health equity a strategic priority at all levels. Increasingly, the health care sector is recognized as having a significant role to play in health equity by creating governance structures and processes to support this aim, tackling the multiple determinants of health on which organizations can have an impact, and recognizing and decreasing institutional racism and other forms of discrimination that sustain health inequities [1–6].

Health equity interventions can be implemented at multiple levels to effect improvements within nations, populations, municipalities, health authorities, organizations, and at the level of clinical practice [1, 7–9]. Specific interventions can vary widely but all are characterized by a common goal of closing the health equity gap with the triple aim of: improving the health of populations, enhancing patient experience and outcomes, and reducing per capita cost of care [1, 10]. In this paper, we focus specifically on a health equity intervention implemented at the level of health care organizations. As we discuss, implementing health equity interventions in health care organizations requires fundamentally shifting taken-for-grant practices, policies and systems that contribute to inequities. This does not imply that health care organizations alone have the power to close the health equity gap; however, they do have the potential to address inequities directly at the point of care, which can affect the determinants that create and sustain health inequities.

Based on our prior research [4, 11], our team designed an organizational-level, multicomponent health equity intervention, titled ‘Equipping Primary Health Care for Equity’ (known as EQUIP) [3], and studied the process and impact of implementing EQUIP in four primary health care clinics in Canada. We purposely selected diverse health care settings so that we could take into account context and complexity [8, 12]. The EQUIP intervention aimed to enhance the capacity of health care organizations to provide care that is explicitly equity oriented. Building on current conceptualizations of health equity [13–18], we defined **equity-oriented** **health care (EOHC)** as an approach that aims to reduce:

- the effects of structural inequities (such as poverty), including the inequitable distribution of the determinants of health (e.g., income and housing) that sustain health inequities;
- the impact of multiple and intersecting forms of racism, discrimination and stigma (e.g., related to mental illness, chronic illnesses, non-conforming gender and sexual identities, etc.) on people’s access to services and their experiences of care; and
- the frequent mismatches between dominant approaches to care (e.g., an emphasis on scheduled appointments versus same day or drop in appointments to accommodate patients’ needs) and the needs of people who are most affected by health and social inequities.

Organizational-level health equity interventions are inherently challenging to implement because they necessitate engaging with issues of power dynamics, racism and other forms of discrimination, and shifts in organizational processes as well as individual staff members’ practices [1, 19, 20]. These foci may not be recognized or valued as part of the core mandate of some health care organizations. Implementing health equity interventions requires a commitment from top-level leadership, dedicated resources to support equity work, changes in organizational structures such as hiring, staff education and care redesign, and tailoring quality improvement efforts to meet the needs of vulnerable populations [1, 5, 6, 20]. Finally, the nature of health care settings - as complex adaptive systems that necessarily shape the uptake of interventions - creates specific challenges [8, 21]. For example, organizational-level health equity interventions can be undermined or supported by structural factors such as policies and funding conditions [22–24]. These dynamics contribute to the fact that upwards of 60% of deliberate attempts at organizational change are ineffective [25–29].

The EQUIP intervention was implemented with four primary health care clinics that served marginalized^1^ populations and, thus, had existing commitments to equity and social justice. The intervention was designed to be tailored to each organization’s unique context, including the local populations served. The purpose of this paper is to illustrate the process and impacts of implementing an organizational-level health equity intervention that aimed to enhance capacity to provide EOHC. We focused on the following research questions: (1) How did staff experience the process of implementation and impacts of the intervention over time? (2) How did the intervention impact the clinics? (3) What are the implications for implementing health equity interventions in health care organizations? In this paper, we focus on the impacts of EQUIP on the staff and the organization. Elsewhere, we report on the pathways between EOHC and patient self-reported health outcomes [30].

## Overview of the Research

Increasingly, leaders in the field of health intervention research are calling for greater attention to studying the process of intervention delivery, and evaluation, in order to understand the intricate human processes that are integral to uptake, delivery, and impact [31–34]. The EQUIP research program provided an ideal opportunity to study these dynamics.

### The EQUIP intervention

The components of the EQUIP intervention are evidence-based, theoretically informed, and guided by a framework identifying three key dimensions of EOHC and ten strategies to support implementation, as described in our prior publication [3]. The key dimensions of EOHC, summarized in Table 1, and the strategies to guide implementation, provided the basis for the EQUIP intervention as described below.

**Table 1:** Framework Guiding the EQUIP Intervention

Trauma- and Violence-Informed Care	• Based on understanding the effects of interpersonal (e.g., child maltreatment, intimate partner violence) and structural (e.g., poverty, racism) forms of violence as intersecting, with compounding impacts on health; recognizes that people disadvantaged by systemic inequities often experience multiple forms of violence that have ongoing traumatic impacts • Shifts emphasis from client disclosure of violence experiences to care providers creating a safe environment, including for those most traumatized • Creates a safer environment for all, including staff; counters the tendency to locate ‘the problem’ of trauma primarily in the psyche of those experiencing violence by emphasizing social and structural conditions as causes of trauma
Contextually-Tailored Care	• Based on understanding how the evolving local community and context shape health and health care inequities • Expands on the notion of patient or client-centered care to tailor services and programs explicitly to the populations served and local contexts • Is responsive to the complexity inherent in designing EOHC with both standardized and tailored components to meet the needs of different contexts and populations; counters a ‘one size fits all’ approach in health care
Cultural Safety	• Based on understanding the impacts of inequitable power relations, racism, discrimination, colonization, and historical and current inequities on health and health care • Shifts attention from ‘cultural differences’ as the source of the problem to the culture of health care as the site for transformation; moves beyond cultural sensitivity to place responsibility on care providers to create culturally safe environments • Foregrounds social justice goals as integral to health care with the aim of shaping health care practices, organizations and policies accordingly

#### Intervention approaches

The EQUIP intervention was implemented between 2013 and 2015. The intervention involved two main approaches: (1) staff education and integration discussions that were delivered via multiple modes over an 8 to 12 month period, and (2) a process of organizational integration and tailoring (OIT) that each clinic led and subsequently implemented over an additional 8 to 12 month period. Recognizing that organizational interventions require engagement over time, the timeline for implementing and assessing the impact of the intervention was intentionally flexible to be maximally adaptable to each clinics’ rhythms, activities, and priorities.

Staff education and integration discussions were implemented at each clinic using a flexible structure tailored to the local context, specific populations served, and organizational priorities, and included a combination of structured face-to-face workshops on specific topics (e.g., trauma- and violence-informed care), general group discussions about issues raised by staff, and online education modules. The specific details of the staff education and integration discussions are described in a prior publication [3] and are summarized here to contextualize the analysis presented in this paper.

The focus of staff education and integration discussions was on: (a) the framework identifying the key dimensions of EOHC and strategies to guide implementation, including the root causes and consequences of health and social inequities, (b) cultural safety education, to explicitly draw attention to institutional and interpersonal racism and other forms of discrimination, and their impacts on health and access to health care, and (c) trauma- and violence-informed care, with an emphasis on strategies to mitigate the health effects of both interpersonal and structural violence.^2^ While didactic educational strategies by themselves tend not to be drivers of behavior change [35], the tailored educational strategies were designed to be relevant to the contexts of these four clinics and used as a catalyst for change in both clinical practices and organizational processes [36]. For each component, a practice consultant facilitated face-to-face discussions designed to help staff connect the content of the education with the specific issues and contexts of their own practices, organizations, and patient populations. This provided a foundation for increased staff awareness, knowledge, and confidence while engaging their interest in enhancing EOHC through practices and policies at the provider and organizational levels.

Organizational integration and tailoring (OIT) was linked to staff education. The practice consultant supported staff to identify the implications of the educational components for themselves, and their organizational policies and practices. The practice consultant then worked with leaders and staff at each site to identify priority areas and develop a unique plan for action. A catalyst grant of $10,000 was provided to each clinic to support practice and policy changes at the organization level. Priority setting for the OIT phase was informed by study-generated ‘Clinic Narrative Profiles’ which included summaries of: (i) the health and social status of each clinic’s patient population with comparisons to population norms (e.g., levels of chronic pain, mental health issues, financial strain, income levels), and (ii) each clinic’s history, and unique sociopolitical and community context. These profiles were discussed with staff to help raise awareness about issues relevant to each clinic (e.g., high rates of chronic pain, substance use, depression, and trauma in the patient population), and revisited during a series of intervention-specific and routine staff meetings, supporting clinic leaders and staff to integrate EOHC strategies and OIT priorities.

#### Settings

While each clinic was unique (see Table 2**)**, a feature common to all four was their commitment to providing team-based care by physicians, nurses and nurse-practitioners, social workers, nutritionists, counsellors, and depending on the clinic, pharmacists, physiotherapists, dentists, and Indigenous^3^ Elders. Patient populations ranged in size from 1300 to 3700. Clinic-based care was complemented with outreach services and programs to varying degrees.

**Table 2:** Descriptions of Each Clinic

Organizational Features	
Clinic A	• Founded in 1994. • Located in a rural region serving farming and First Nations communities. • Provides primary care at multiple sites to populations across the lifespan, from seniors to families with young children, through direct primary care and a wide range of responsive health promotion programs.
Clinic B	• Founded in 2011. • Located in a city which is a regional hub for many rural communities. • Serves people who face barriers to health and social care and those ‘in transition,’ with a primary focus on women and families living in marginalizing conditions, including recent immigrants, many of whom have experienced violence and trauma. • Primary health care services include identification, ongoing assessment and management of acute and chronic health problems, counseling, education and health promotion, and support in navigating complex systems.
Clinic C	• Founded 1970. • Located in an inner city metropolis and serves low income populations, including many experiencing inadequate housing or homelessness, major mental health and substance use issues, and significant barriers to accessing basic health services. • Provides a wide range of primary health care services, including a pharmacy, dental clinic, and physical and mental health services; operates on the basis of a 'Housing First' service philosophy.
Clinic D	• Founded in 1991. • Located in a northern regional city where high proportions of Indigenous peoples reside. • Serves Indigenous and non-Indigenous peoples experiencing major socioeconomic challenges including people living on very low incomes, in unstable or temporary housing, and those who are unable to work due to disability. 75% of the patient population self-identifies as Indigenous. • Provides a wide range of primary health care services including medical and nursing care, counselling, social work, physiotherapy, and outreach services.

## Methods

We used a transformative embedded mixed method design [37, 38] to examine the impact of the EQUIP intervention on the clinics’ organizational processes and priorities, and staff members’ knowledge, practices and confidence levels related to EOHC. Mixed methods approaches are well suited to understanding complex patterns of organizational change across varied contexts and, consistent with this study, can be employed in ways that reflect social justice and equity aims [38]. In this analysis, we drew on three types of data collected concurrently between 2013 and 2015:

- a brief quantitative survey of staff members’ confidence with regard to providing EOHC before engagement with the intervention (baseline), which also served as a needs assessment, and which was repeated to gauge staff members’ confidence levels related to EOHC during the intervention, and after the intervention was implemented;
- in-depth, open-ended interviews conducted with individual staff members at the conclusion of the intervention, which focused on their experiences engaging with the intervention and any perceived impacts (positive and negative) on their practice, and on the organization;
- observations in each setting of the general milieu and more detailed observations of staff meetings recorded as fieldnotes.

While we expected that all data would help inform our understanding of the nature of change within the clinics and the impacts of the intervention on staff, in this analysis, the qualitative data were positioned as primary and central to developing an in-depth understanding of the process and impacts on staff in ways that privileged their voices. The survey data were combined with the qualitative findings to extend and contextualize our understanding of impacts on staff confidence levels.

### Data collection

#### Staff survey

Prior to the baseline survey, a letter of information was distributed to staff by email. Those who wished to participate created their own anonymous identification number and completed an informed consent process online. Five items from the survey assessed staff confidence related to EOHC and are used in this analysis to help contextualize the qualitative findings. These items were generated by the research team, with the format modelled after recommendations of Bandura [39] to capture confidence (self-efficacy) after an extensive search of the literature revealed that no similar items existed. The items were pre-tested with staff working in a primary care clinic that was not included as a study site. Based on their feedback, items were revised to enhance clarity. Staff were asked to rate their confidence in a number of domains on a 10-point Likert scale ranging from ‘*not at all confident* (1)’ to ‘*completely confident* (10)’. The staff survey was repeated 12 months later (at Time 2, following the staff education components of the intervention), and 24 months later (at Time 3, after completion of the OIT phase of the intervention) to monitor staff confidence over time.

Given our interest in using these data to describe trends at the organizational level (rather than individual level), and knowing that there would be staff turnover during the 2-year implementation period, we did not restrict participation of staff to those who completed the baseline survey, but encouraged all staff to take part. The identity of participants was not known to the research team. At all three data collection points, staff members who were employed in a variety of roles participated, including physicians, nurse practitioners and registered nurses, medical office assistants (MOAs, who are front-desk reception staff who greet patients, schedule appointments, manage the waiting room environment, and field patients’ questions), social workers, and other providers such as pharmacists, outreach workers, nutritionists, and administrative staff. Thus, the staff survey sample size varied at each time point (*n* = 86, 82, 57, at Times 1, 2 and 3, respectively).

#### In-depth interviews

At each site, staff were purposively invited to participate in interviews to maximize diversity across the various staff roles within each clinic. Thirty-one staff members from the four clinics participated including 5 registered nurses, 5 nurse practitioners, 3 physicians, 4 clinic leaders, 5 social service providers such as social workers, counselors and outreach workers, 3 MOAs, 3 administrative staff, and 3 staff in other roles (e.g., pharmacy, nutritionist). The interviews were conducted primarily by the principal investigators, who had extensive experience conducting qualitative interviews, with some interviews conducted by trained research staff. Most interviews took place in quiet, private spaces at the clinic, and at the request of some of the participants, several were conducted outside of the clinic. Interviews lasted from 30 to 60 min, with the interview questions focusing on: staff members’ experiences engaging with the EQUIP intervention; perceived impact or lack of impact for themselves, their practice, patients, or their organization; and how their organizational context may have influenced engagement, uptake or impact of the intervention. All participants completed an informed consent process and the interviews were audio-recorded and transcribed verbatim. All identifying information was removed prior to analyzing the transcripts.

#### Observations

Observational data recorded as fieldnotes provided important contextual information regarding the process and impact on staff and the organization of the EQUIP intervention. Approximately 380 hours of observational data were collected by the principal investigators, who are experienced ethnographic researchers, and the practice consultant who engaged with all four sites throughout the intervention. Observations occurred at key points including: at team meetings during the outset of the intervention, during time spent in the clinics observing the general milieu, and during the staff education and integration discussions to track staff reactions to intervention activities.

### Data analysis

An interpretive thematic analysis of the interview and observational data was conducted [40, 41] using NVivo for organizing and coding the data. Data were read repeatedly to identify recurring and contradictory patterns, and possible linkages to theoretical constructs. Observational data provided a means of integrating interview data and staff survey data in the analysis, enhancing rigour. As data were analyzed, coding categories were refined and the analysis shifted to a more conceptual representation of themes pertaining to the impact and implications of engaging with EQUIP as an organizational-level health equity intervention.

Staff survey data were analyzed using descriptive statistics by time period, including data from all staff who completed the survey at each time. These data provide a snapshot of trends in staff confidence within each clinic as a whole. Since we did not focus on testing individual changes over time using matched cases, no tests of significance were performed. These data were integrated into the qualitative analysis as a general point of departure from which more nuanced findings about the impacts of EQUIP on staff over time were positioned.

## Results

**Fig. 1:**
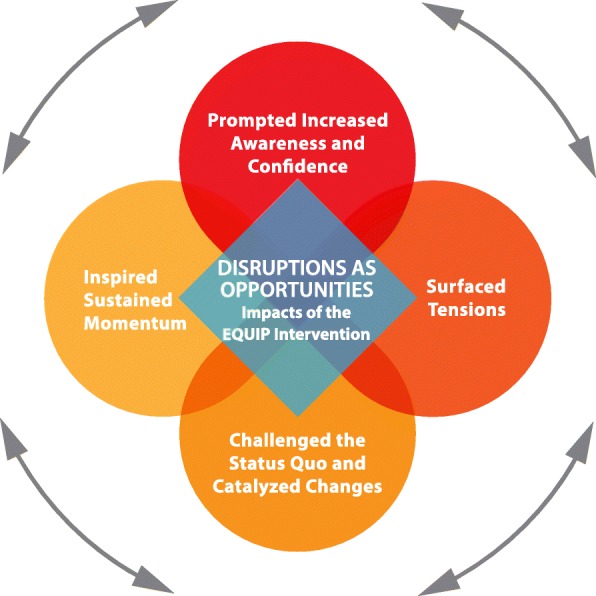
Disruptions as Opportunities

As the clinics engaged with the intervention, and as staff members’ awareness and confidence related to EOHC shifted, tensions that often pre-existed in the organizations were surfaced, prompting efforts to challenge the status quo and catalyze changes that the clinic staff and adminstrative leaders then worked to sustain – some of which exposed further or recurring conflicts. These impacts are depicted in Fig. 1 below.

### 1. EQUIP prompted increased awareness and confidence

Overall, staff reported high levels of awareness of EOHC and related issues prior to the intervention, in large part because the clinics had explicit mandates to foster equity. As expected, staff described different levels and types of impact, reflecting their own level of commitment, understanding, and varied social and professional positions. Some who perceived their organization as already equity-oriented described how the intervention had a limited impact, and yet it provided an opportunity to build on their level of awareness and enhance their practice: “all those things existed before but they’ve been…embellished.”

As shown in Table 3, on the survey, staff reported an overall increase in confidence related to specific aspects of EOHC over time. While baseline scores for confidence ranged from 5.3 to 5.6 out of a possible 10, these scores all increased to more than 7.0 (range 7.0 to 7.7) post-intervention. Notably, at completion of the intervention, when asked how much their confidence to provide EOHC had changed over the previous 2 years, 43 of the 56 staff (77%) who completed the final survey reported that they were much more confident, while 23% reported no change in confidence; no staff reported a decline in confidence.

**Table 3:** Staff Ratings of Confidence in Selected Aspects of Equity-Oriented Health Care at Pre-Intervention, Post-Education, and Post-Intervention

Items	Pre-Intervention (*n* = 86)^b^	After Staff Education (*n* = 82)	Post- Intervention^c^ (*n* = 57)
In a patient encounter, how confident^a^ are you that you can:	M (SD) n	M (SD) n	M (SD) n
1. explain to a patient what trauma is?	5.4 (2.39) 64	6.6 (2.28) 65	7.2 (2.10) 48
2. explain the effects of trauma to a patient?	5.3 (2.32) 64	6.5 (2.40) 65	7.2 (2.29) 49
3. recognize the signs and symptoms of trauma, even if a person does not verbally tell you that they have experienced a traumatic event?	5.7 (2.36) 68	6.3 (2.36) 69	7.0 (2.16) 51
In general, how confident are you in your ability to effectively deal with biases, discrimination, and prejudices in the clinical setting?	5.8 (2.02) 76	6.9 (2.00) 77	7.7 (1.34) 56

^a^Based on a 10-point Likert scale ranging from ‘*not at all confident*’ (1) to ‘*completely confident* ‘(10)

^b^Sample size at each time point and for each question varies due to staff turnover, missed responses, and the voluntary and anonymous nature of participation

^c^After completion of the Organizational Integration and Tailoring (OIT) phase of the EQUIP intervention

For some, the impact of the intervention on their overall awareness of the root causes of health and social inequities was described as intense. One staff member, an MOA, said:

I feel like I’m a very open person but I just didn’t realize all the things that have kind of happened, right? So going through all that [the EQUIP intervention], I think personally for myself, has made me be even more accepting of our clientele…. And I think I already was, but even more so now where I just want to try and do that extra thing for them. And I get very frustrated when walls are put up, you know, from the system, it’s the system.

In another clinic, a member of the leadership team described the impact of EQUIP as follows:

The expectation is that people will come with a willingness to work in a setting like this, and these values of equity and these things are much more identified. We used to have it, you know, we assumed it was there; now it’s going to be more solidified for us.

One of the physicians also explained how EQUIP led to critical shifts in self-awareness: “the only real way that I have to change the system is to look at myself first.”

Staff members realized that their organizational mandates to pay attention to equity were somewhat ‘out of step’ with values driving the larger health care system, and that the positioning of the clinics within the wider health care system limited their possibilities to provide EOHC. As this administrative leader described:

The system hasn’t moved fast enough to get ready for the complexity of the clients coming, (...) and now they’re just starting to panic (...) throw everything out of the hospital without any kind of time to develop [community-based care]....There’s just cuts and then it’s gone. ... And no infrastructure for community to grow and deliver anything else, right? Aging buildings, no surplus, lack of retention of staff, it’s just endless, our unpreparedness for what’s coming.

At the beginning of the intervention, providing the staff and leaders with a study-generated ‘Clinic Narrative Profile’ -- which included their community and clinic socio-historical context, and demographic and health status data on their patients compared to population norms -- sensitized staff to the interconnectedness of chronic pain, depression, violence and trauma, and substance use, which were impacting large proportions of patients. As explained by this staff member: “to have those kinds of [health and social status] figures in black and white and to see the amount of trauma, to see the levels of mental health issues is very valuable.” The intervention therefore helped staff and administrative leaders to target certain areas specific to each clinic’s context and to the specific populations served.

To summarize, staff reported shifts in awareness and increased confidence regarding EOHC as an outcome of the EQUIP intervention. The magnitude and nature of these shifts varied and the process underlying these shifts raised some specific tensions, including both intrapersonal awareness, and an awareness of organizational and system-level barriers (implicit and explicit) to EOHC.

### 2. EQUIP surfaced tensions

EQUIP drew attention to issues at the heart of health inequities, such as the ways racism and other forms of oppression operate at both the individual and system levels, and how power imbalances shape organizational processes and care practices. EQUIP made these issues more visible to leaders and staff, and created opportunities to explore individual and organizational challenges, and to reflect upon and discuss differences among staff. One staff member noted:

We do need to look at our own practice; we do need to look at the sensitivities and the barriers that we still do put up. Because of…having and doing all this work [the EQUIP intervention], I think that we are more honest towards ourselves, more open to challenging ourselves and each other, to say, are we on the right path?

Not surprisingly, EQUIP revealed pre-existing issues that were salient to each clinic. These pertained to the need for better ways to address: (a) the continuities between structural violence and patients’ lived experiences of trauma and violence; (b) the negative impacts of systemic and interpersonal racism and other forms of discrimination on patients and their families; and (c) how to better support patients with substance use issues, especially when there were poorly defined or conflicting approaches.

As reported elsewhere [30, 42], rates of poverty, posttraumatic stress disorder, depression, and chronic pain in the clinic populations were very high compared to population rates. These baseline data were reported back to the clinics at the outset of the EQUIP intervention, and during the staff education sessions about trauma- and violence-informed care, emphasizing data that showed the intersections among structural forms of violence (e.g., institutional racism, policy-induced poverty) and interpersonal forms of violence. Discussing the health effects of trauma and violence uncovered how both individual practices and organizational policies impact patients, and made staff aware of the need to be more responsive to high rates of trauma. They gained awareness of how to avoid potentially re-traumatizing patients in unintentional ways, as this staff member explained: “The trauma- and violence-informed care…that one helped me to think….about how you could re-traumatize someone with a simple action.” At each clinic, discussions about trauma and violence also made more evident often unspoken concerns about compassion fatigue and vicarious trauma, which opened up discussions about the staff’s emotional well-being. This physician, a long-term employee, described their growing awareness as follows:

I was pretty burnt [last summer] -- not really happy with the way things were going and frustrated that it’s the same thing over and over and over. How do you find what you need to continue doing the work in those circumstances? … I’m wondering, am I cut out for this?

The cultural safety component of the intervention was particularly powerful in surfacing tensions related to staff members’ increasing awareness of the pervasiveness and negative impacts of systemic and interpersonal racism, and the role and responsibility of providers and organizations in responding to racism. Some staff saw their own practice as deeply transformed by the cultural safety component of the EQUIP intervention, which had a focus on strategies that could be used to counteract racism and discrimination in relation to Indigenous peoples, and offered insight into how these processes shape health and health care experiences for other groups experiencing racialization, stigma and varying forms of discrimination. As this staff member described:

That’s probably the largest impact. It’s just being more aware….I hadn’t even heard of that [term] ‘settlers’ before. So that was interesting because I kind of realized how I may be perceived…So, I think that was the largest impact, just how I’m seen, even if I’m not saying anything, how I just might look.

In other cases, the focus on racism and discrimination drew attention to inconsistencies between the equity-oriented mandate of the clinics and the stance of some staff. In one clinic, the dynamics arising from these discrepancies resulted in a staff member being asked to leave the organization because of irreconcilable differences about strategies to respond to issues of racism. This exacerbated the pre-existing high staff turnover, as this administrator described: “it’s more than fifty percent turnover. …And it will be more, it will be more before this settles out. I would say we’re going to be up around 75%.” As discussed below, these areas of strain, although unanticipated, resulted in organizational shifts to promote greater attention to the fit of staff with organizational mandates, and organizational policies to more adequately support staff.

One unanticipated issue was in relation to substance use, which while not initially conceptualized as an explicit focus of the EQUIP intervention, was an area in which challenges arose as each clinic considered how substance use intersected with high rates of trauma, violence, chronic pain, economic disadvantage, and mental health issues. For example, conflicts were revealed between staff and clinic leaders who were trained in abstinence approaches to substance use, and those who were committed to reducing the negative effects of substance use without promoting abstinence. Discussions among staff ensued in relation to patients judged to be ‘drug seeking’, and with regard to how to respond to difficult behaviors when patients were under the influence of substances. As discussed below, each area of tension provided a focus for challenging the status quo, and for innovative shifts in provider and organizational practices.

### 3. EQUIP challenged the status quo and catalyzed changes

The EQUIP intervention led some staff within the clinics to challenge the status quo, and in some cases led other staff to resist such challenges. These challenges were opportunities for taking risks and catalyzing changes. These changes occurred in three key areas, reflecting the areas in which organizational tensions arose around how to more adequately respond to the impacts of a) trauma and violence, b) racism and other forms of discrimination, and c) intersecting issues related to substance use, trauma and chronic pain.

#### a) Responding more effectively to intersecting forms of trauma and violence

The specific education and integration discussions on trauma- and violence-informed care foregrounded the notion of structural violence, a concept that was new to many of the participating staff. Uptake of this component of the intervention was reflected in changes to the usual discourses used in team meetings to contextualize the broader historical and economic conditions shaping patients’ health issues, and the approaches proposed for supporting patients. For example, at one of the clinics that served high proportions of Indigenous peoples, many of whom experienced significant individual and systemic traumas, the language, perspectives and tone used to describe patients’ histories and clinical conditions shifted markedly during EQUIP. Specifically, staff made efforts to limit the dominance of biomedically-based discourses in team meetings, so that patients’ community and socio-historical contexts might be discussed and factored into decision-making regarding their clinical care. Fieldnotes captured how team members attempted to contextualize patients’ lives and social contexts. For example, instead of discussing “a 58 year old First Nations woman, alcoholic, history of child abuse, refusing Hep C treatment”, we were more likely to hear “This 58 year old woman is from [community name]. She is a residential school survivor, and has had a very challenging life. She has problems with alcohol when her son is living with her, and is very worried about side effects if she takes Hep C treatment” (Fieldnotes). During team meetings, this required staff to limit the ‘air time’ given to physicians and nurse-practitioners, and actively invite and encourage input from other staff members, including receptionsts, outreach workers, social workers, nurses, counsellors and Indigenous staff members. Shifts in team meetings and in staff dynamics were observed and discussed by staff:

In the meetings, it’s starting to shift, which is really big, because for years, we’ve been saying, ok, we need the psychosocial piece to come out in the meetings and not talk three quarters of the time about the medical stuff.

In the process, challenging the status quo meant observable shifts in power imbalances that previously existed between those having a biomedical orientation (particularly, physicians and nurse practitioners) and those with a more holistic orientation (such as counsellors, nurses or Elders) or non-medical orientation (such as social workers and outreach workers), as well as between reception/clerical staff and clinical staff.

In each clinic, the staff education and integration discussions led to increasing recognition about the impacts of patients’ everyday experiences of structural violence, and in particular, resulted in MOAs reconsidering how routine clinic processes could be problematic. In team meetings, MOAs framed the practice of conveying a dismissive tone to patients over the phone as “examples of structural violence”, prompting them to shift their phone protocols and etiquette. In an effort to create a less adversarial and more welcoming waiting room in one of the clinics, the MOAs advocated removing the large (and only) sign at the reception desk that warned patients to “check in or you will lose your appointment.” The MOAs also engaged in a process of reconsidering a policy requiring patients seeking same day appointments to line up on a busy thoroughfare outside the clinic to wait for the clinic doors to open. They expressed an increasing concern about the extent to which this policy exacerbated patients’ experiences of feeling judged negatively or harassed by people walking or driving by in the surrounding neighborhood. As a result, the MOAs made a case to the clinic leaders and other staff to permit patients to wait inside the clinic instead of on the sidewalk. One MOA described the impact on clients:

They’re definitely more relaxed in the morning so that would make them, you know, feel a little happier and more comfortable. Just to have a place to come in and relax and not have to stand outside in a lineup on the street. So it sort of changes just like that concept of, you know, lining up like cattle.

#### b) Responding to the impacts of racism and other forms of discrimination

Among the most impactful effects of engaging with the EQUIP intervention were the shifts experienced by staff members with regard to their own understanding, confidence, skills and practices related to issues of racism and other forms of discrimination. Several described increased confidence to be able to address racist discourses encountered in their communities or within their places of practice:

I thought, you know what, by not saying anything, that just makes me just as guilty. So in a lot of social situations, if something comes up negative about, especially First Nations people, I’m much more vocal.

The cultural safety component of the intervention was influential in a number of unique ways – not only in relation to working with Indigenous peoples, but also in relation to working with new immigrant populations. In one clinic, staff focused their discussions on cultural safety in relation to trauma- and violence-informed care in light of the high levels of trauma experienced by the refugees they were newly serving, and on how to better serve this population, as this staff member explained:

And now I’m realizing that a lot of our immigrant population that we are seeing are coming as refugees, and that [the key dimensions of equity-oriented care] apply just as much… One fellow in particular who presented with acute depression… from the Congo….I don’t need to know what happened to him when he was in hiding to recognize that even a discussion of family put him right back there.

In interviews, and as observed during conversations as EQUIP was implemented, staff noted that the EQUIP components drew attention to the racialization of inequities, at times provoking defensiveness and efforts to minimize conflict, and sometimes provoking renewed engagement in efforts toward equity. In some cases, staff members expressed discomfort with the cultural safety component of the intervention, reflecting what they described as an internal struggle related to the emphasis on acknowledging historical trauma as integral to reconciliation:

I know maybe this is my own lack of comfort, so maybe this is my own kind of conviction: I found some of the things on colonization really difficult to watch on the video. And I sometimes wondered if there was too much emphasis on the past because you can’t change the past. You can learn from the past and you can predict a change for the future but it’s kind of like alright, I’m the bad guy, I know it, so you know what I mean? [Laughter] What can we do now, you know what I mean? Because some of my clients are stuck in the past right, and I know that’s not their fault, that’s not our fault, that’s just where they are at this time, that timeline of life right?

EQUIP’s attention to inequities including racism and poverty concurrently exposed power inequities and strained dynamics among staff related to professional hierarchies as well as societal inequities based on gender, ethno-cultural heritage, substance use issues, and sexual orientation. In two clinics, attention focused on whose voices among the Indigenous and non-Indigenous staff were privileged in decisions shaping clinical practices or programs:

It’s been a catalyst, an awareness of potential relation problems between us and [the neighboring Indigenous community] that we didn’t even know were there, or I didn’t know were there. And what can we do about that? How can I be always mindful of that?

To address these and related concerns, clinical and administrative leaders at this clinic worked to develop a relationship with the local Indigenous communities:

We work with [names] and [name] is also in charge of all the clients receiving homecare in the community, so now they communicate with us….At first it was kind of standoffish. Now I feel like we’ve actually been really embraced by the [Indigenous community] team, and that [names] call us all the time. We have group meetings with them, we do rounds with them.… It seems to have bridged a really strong relationship just from the front-line workers' perspective. And I think that is really, really helping, and I feel really accepted and embraced.

Other staff at this clinic acknowledged that the effort to establish effective working relationships is ongoing, emphasizing that change was happening, but that “some of the people that had the mistrust still have the mistrust.”

At another clinic, the OIT priority became hiring an Indigenous Elder who could be integrated into the clinic’s activities, for example, within the waiting room to talk informally with patients who wanted to speak with an Elder; to participate in interdisciplinary team discussions as a way of featuring more attention to the socio-historical context of patients’ lives; and to be available to patients who requested one-to-one counselling sessions with the Elder. The increasing awareness regarding issues related to Indigenous peoples was important, however, the clinics also attended to a range of forms of discrimination, including racism associated with anti-immigrant sentiments, gendered inequities, and stigma associated with mental health issues and substance use.

#### c) Responding more adequately to intersecting issues related to substance use, trauma and chronic pain

The third area in which the status quo was challenged, was in relation to substance use. In each clinic, debates arose regarding the merits of abstinence or harm reduction^4^ approaches, and how to structure clinical practices and organizational policies to optimally serve people with substance use issues. In some cases, discussions were framed explicitly in terms of these different philosophical approaches, whereas in others, they were implicit or not necessarily recognized. For example, one clinic had a stated commitment to harm reduction, but staff reported not knowing what that meant. In another clinic, a physician described feeling “gob-smacked” upon learning from a different clinic about their harm reduction strategies:

I mean the harm reduction concept is so much bigger than I even understand, I’m still struggling with some aspects of it. So [EQUIP] really sort of took it to another level for me. So that opened my eyes…My initial response was like “Are you nuts?” And my second response was “well wait a second, listen to the reality that they’re dealing with, number one, and what are they doing?” They’re trying to engage this person and if they succeed … maybe this person will sort of come with them over to the clinic and have some blood work or I don’t know what.

A high proportion of the clients experienced both substance use and chronic pain issues, so pain management was a key challenge. For example, fears among prescribers about misuse of opiate or other prescription medications prompted often heated debates about the ‘contracts’ that some patients were required to sign regarding the use of controlled medications. Understandably, given different professional and legal responsibilities, disagreements often arose between providers from different professions, and a key challenge was how to prescribe opioids. Fieldnotes documented the negotiations among staff, within teams, and with patients in this area, and highlighted the complexities of prescribing as this staff member discussed: “People have their own philosophies about how you manage pain [via pain medications] and suffering…their philosophies are based on decades of experience as well as their own personal values and beliefs.” At one of the clinics, the EQUIP intervention led directly to the development of a harm reduction framework and policy:

So I think the coming together of a common set of values or beliefs .... We’ve always had the community-health clinic philosophy but now …… we believe in harm reduction. And that, to my understanding, is also going to be part of the orientation from now on. Like when I first started here…there was nothing on cultural training, there was nothing on harm reduction…So now there’s more awareness and shifting towards that, I think, overall as an organization.

Staff acknowledged that adopting a focus on harm reduction would require ongoing discussions within their organization, and that divergent views would continue to become apparent. Explicitly naming harm reduction as an organizational commitment represented a bold step for this clinic, and involved offering a series of sessions on harm reduction that engaged groups of staff, board members and Indigenous partners, facilitated by the EQUIP practice consultant. The clinic administrator described the impact:

The staff here has amazed me in the way that they have taken it on and said yes to these [commitments to integrate harm reduction]. It’s certainly not been a hundred percent. I think what it’s done is also raised tensions as far as what does it mean with our difficult, challenging clients. What does that mean: ‘if it's harm reduction does that mean that we just give everybody what they want?’…So, what I think has percolated up is a lot of discussion in different pockets. And then I think I see people trying harder to check their judgment. And I hear more people saying, you know, look at their [patients’] context….So I don’t know that everybody is completely on board or on side but…we all are trying to work in that direction.

As the OIT processes unfolded, each clinic identified priorities for change that reflected the underlying tensions, challenges and changes that had been identified.

### 4. EQUIP inspired sustained momentum

Each clinic initiated and led activities designed to enhance EOHC. These were initiated under the auspices of EQUIP but continued to evolve beyond the duration of the intervention, and are summarized in Table 4. Staff and leaders recognized that these activities required ongoing effort.

**Table 4:** Activities Designed to Enhance Equity-Oriented Health Care

	Examples of Key Equity-Oriented Actions
Clinic A	• Initiated new working relationship with the local Indigenous community in an effort to address the history of tense relations between the Indigenous and non-Indigenous communities, leaders and staff members. • Developed new harm reduction policies and practices to reduce stigma and enhance the range of strategies for supporting patients with substance use issues.
Clinic B	• Integrated explicit trauma- and violence-informed approaches into the routine provision of care in part in response to the influx of new immigrants and refugees with significant histories of trauma and violence. • Developed a strategy for preventing and responding to vicarious trauma experienced by staff.
Clinic C	• Opened the clinic doors 30 min earlier so that patients could wait indoors to book same-day appointments, instead of lining up outside the entrance exposed to the elements and judgements of passersby. • Initiated a chronic pain group to help patients learn about the often complex causes of chronic pain and non-pharmacological pain management strategies.
Clinic D	• Repurposed a section of the waiting room to create a child-friendly space for women caring for small children as a strategy for promoting safety and comfort. • Hired and integrated an Indigenous Elder to participate in clinic activities including visiting with patients in the waiting room and participating in clinical team meetings to enhance incorporation of Indigenous knowledge and approaches.

Sustaining momentum beyond the research process required both leadership and resources. Each clinic varied in these elements. Across all clinics, staff consistently recognized the importance of engaged leadership to make EOHC possible, as this leader/administrator explained:

It’s not education as much as helping to develop a culture, a culture where staff are open to sometimes examining themselves, but also of feeling everybody has a voice to some degree within that, within the organization. So it’s a style of leadership, but the style of leadership also perhaps develops the culture.

For many staff, the responsibility for sustaining movement toward enhanced EOHC lay with the immediate administration; others recognized that leadership decisions were shaped by larger resource and structural conditions, and policy and funding pressures. Some staff in the various sites also felt disconnected from the board members or administration, for example, expressing concern that their workloads and the vicarious trauma they experienced as a consequence of continuously providing care to people facing high levels of violence and trauma, were not recognized or dealt with. Staff members (from three different clinics) remarked:

I’m not convinced that administration has any concept of how swamped we’re all feeling.

This lack of communication from the board, and this is what I’m hearing, and I’m kind of seeing it, this lack of involvement from the board has caused … low morale.

Sometimes I feel like there’s a bit of a disconnect between the primary care providers, like what happens down here, like providing care for the patients, and then what goes on upstairs [in the administration area]?

In one clinic, staff described how the entrenched leadership patterns meant that the intervention could not achieve its full effect:

We felt that there were changes that should be made within the leadership but it’s like the leadership wasn’t able to do that…That’s probably the fear. We opened this up…This is a very good place to work, don’t get me wrong…but there are things that happen that are within the leadership and how it runs that we don’t feel like is always the best for everybody.

In all cases, however, leaders and staff expressed growing awareness of the time required to respond constructively to tensions, and that often, changes unfolded in slow, non-linear ways:

I mean the change has been slow and gradual so it’s kind of hard to really see because what it also does, I think, it upsets the apple cart… I think with this trauma stuff and the other stuff, it’s like, it brings out the issues I guess, it brings out the issues where you talk about them and maybe deal with them, but there’s other stuff it brings out.

Importantly, focusing on the impact of structural and interpersonal violence on patients’ lives illustrated the need for viable and sustainable ways to support staff experiencing vicarious trauma stemming from their work with populations who experience high levels of violence and trauma. Three of the four clinics realized that they did not have robust employee assistance programs to support staff dealing with vicarious trauma. At one clinic, leaders and staff prioritized a process for working together on previously unacknowledged trauma that many were experiencing, focusing their OIT activities on team-based strategies and supports to better address vicarious trauma.

## Discussion

The process of implementing EQUIP has extended our understanding of the impacts of an EOHC intervention on staff and the organization, the organizational tensions that can surface in the process of engaging with such an intervention, and the structures and supports needed to manage the challenges and opportunities that can arise. EQUIP demonstrated that low-cost, high-impact strategies can enhance the delivery of equity-oriented care. An over-arching theme arising from our analysis of the impact of EQUIP on staff and the organizations is that of disruption as opportunity.

Recently, interventions to improve health and health care delivery have been critiqued as being too conservative, and not disruptive enough of the factors that entrench health inequities [8, 21]. To counter this tendency, Hawe and others are calling for interventions that are “maximally disruptive” to bring about change in deep-seated patterns and conditions [21]. Such interventions require supportive and often new organizational structures and processes to manage the shifts in work, power dynamics and taken-for-granted practices and processes [1, 21]. Interventions need to be designed to move beyond more customary methods (e.g., educational workshops) by changing the dynamics of the settings that structure routine patterns within organizations [21]. In addition, mechanisms and policy directions are needed to optimize the health care system's capacity to support EOHC [43]. 

As observed in the EQUIP study, ongoing dialogue and accounting for inter-professional power dynamics are key to converting abstract ideas about health equity into meaningful, concrete actions in busy, complex clinics. Leadership and resources (e.g., staff time) are needed to anticipate areas of potential conflict, and to identify ways to respond constructively, including to the often difficult conversations that arise. Disruptions can reveal opportunities for enhancing health equity. For these opportunities to be realized, clinic leaders and staff must share a vision of the potential positive outcomes. Focusing on the positive and productive aspects of change can help preempt staff members’ or leaders’ sense of “it’s a good idea, but it’s too hard” in reaction to calls for EOHC. Linking the notion of positive disruption to concepts such as innovation, ingenuity, opportunity and interdependence could be important to prompt an organization’s sense of applying a collective will to intractable problems for a collective benefit; focusing on the enhanced confidence of staff can bolster this sense of ‘can-do’. The findings from the EQUIP study demonstrate that tensions can be helpful and result in positive organizational shifts that have potential for high impact, particularly when working with patient populations who experience significant challenges in accessing care. The extent to which the EQUIP intervention was effective in increasing staff confidence about EOHC and shifting practices that operate to curtail EOHC, was enabled by the following key features:

- The intervention involved all levels of staff. In each clinic, people who may not typically be included in staff-wide education, such as receptionists and MOAs, were key to the initiatives undertaken. Further, dialogue among various categories of staff was central to both identifying and working to resolve areas of tension.

- The intervention components were powerful and impactful. Feedback from the education and integration sessions, and survey results, indicated that the staff gained knowledge and strengthened their commitment to EOHC, especially those who were relatively less powerful within their clinic.

- Narratives about the socio-historical context of the local communities/populations served, and data feedback loops were effective in helping the clinics prioritize their OIT needs and ongoing needs for sustainable change.

Findings from the EQUIP study help to illuminate the complexities involved in implementing organizational-level interventions, even in settings that have an explicit commitment to serving populations who experience health and social inequities. Further research is needed to study the implementation process and impacts in primary care settings that may be more mainstream and in health care contexts that place less emphasis on the need to address health equity issues, but also serve marginalized populations (such as emergency departments or mental health settings). Although clinic leadership and staff were committed to participation, the high levels of turnover and the length of the study meant that the sample size for the survey varied and included some different people at each time point, preventing an analysis of changes in confidence at the level of individual providers. This challenge may be exacerbated in settings with less explicit commitments to equity. Studies that prospectively track a more comprehensive set of staff, organizational and patient outcomes are needed to more systematically understand the impacts of introducing EOHC in clinical settings, and to identify factors that contribute to success across varied contexts. Findings from EQUIP point to the types of outcomes that should be measured, and to practical issues that should be considered in designing these studies.

## Conclusions

**Fig. 2:**
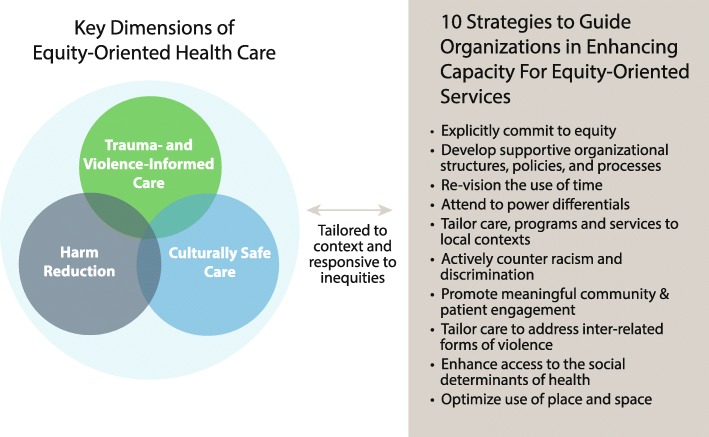
Key Dimensions of Equity-Oriented Health Care and Strategies to Guide Implementation

While the EQUIP intervention surfaced tensions that led to particular disruptions and actions, our analysis of the process also suggests ways that the intervention could be enhanced to have greater impact. We, therefore, recommend the following for implementing equity-oriented interventions:

- *Prepare for disruptions.* We suggest framing interventions as necessarily disruptive, with an emphasis on the positive and productive aspects of such changes, the potential impact of small, low-cost shifts in organizational practices, and transparency about these as intended parts of the process. In retrospect, the areas of disruption were predictable, suggesting that we could have anticipated them collaboratively with the clinics. Consequently, we suggest that an equity-oriented ‘strengths, weaknesses, opportunities and threats’ (SWOT) analysis be undertaken by staff and leaders as a first step in any equity-oriented intervention. Anticipating these areas will provide guidance for change management. Staff will require support to effectively deal with issues that will inevitably arise. For example, drawing on anti-racist/anti-oppression approaches could provide guidance as to how to anticipate and pre-empt defensiveness engendered in addressing racial privilege, which is a necessary part of the process of working toward cultural safety. Shifts in organizational policies to support the implementation of harm reduction strategies would be one way to address the intersecting impacts of structural violence and trauma on patients’ lives.

- *Involve all staff.* To spark deliberations about feasible and implementable shifts in practices or policies that can enhance EOHC, it is essential to involve staff from all kinds of roles and job categories. Seeking staff perspectives, even on ostensibly small changes, is critical to sustaining changes. In this study ‘non-clinical’ staff made some of the most profoundly impactful changes. We recommend intensifying and expanding this inclusivity (e.g., through explicit engagement of board members, MOAs, auxiliary staff or others who might not be routinely seen as ‘staff’), and make the importance of doing so clear at the outset.

- *Maximize opportunities for ownership of the intervention*. In an effort to minimize the burden of the research on staff and leaders, the EQUIP research team and practice consultant coordinated much of the intervention activity. This did not optimally build ownership, or garner the maximum input from the clinical sites. We suggest that leaders and clinicians should have as much ownership and control as possible, including, where feasible, delivering the intervention activities. A staged approach to EOHC could be adopted, in which clinics or departments identify the areas of learning/skill-building where they might start, and what would be a productive entry point (e.g., building on existing strengths); they could develop their own learning plan/trajectory to suit their context and staff needs. The resources to lead all aspects of the intervention may not be available in all settings; whether internal or external, people with facilitation skills and expertise in EOHC will be an asset.

- *Pace the intervention for intense delivery over a relatively short time frame*. While flexibility regarding the timing of the delivery of the EQUIP intervention reduced the burden on the clinics and fostered collaboration, staff routinely commented on the extended time between components, and that they ‘lost track’ of the intervention. ‘Tune-ups’ may be required over time to ensure retention, particularly if the bulk of the intervention is delivered in an intense burst, or if there are long intervals between activities.

- *Strategize patient involvement.* Given that patient involvement is fundamental to equity-oriented care (e.g., cultural safety depends on patients identifying what safety is to them; patient engagement is foundational to trauma- and violence-informed care), equity-oriented interventions require strategies to foster patient involvement, such as the formation of patient advisory committees or other means of seeking meaningful input.

- *Explicitly integrate harm reduction as a key dimension of EOHC.* The key areas of disruption that emerged were related to a) trauma, including vicarious or secondary trauma for staff, b) racism, and c) substance use. Our team had anticipated and planned for the first two areas, providing staff education related to trauma- and violence-informed care and cultural safety. However, staff identified approaches to substance use as problematic in all clinics. Although we were aware of this before the intervention in at least one clinic, we did not anticipate how significant the problem of a lack of an equity-oriented approach to substance use would be. Recognizing this led our team to reexamine the data upon which we based our initial conceptualization of the key dimensions of EOHC. Based on that review and our experience conducting the EQUIP intervention study, we propose that an equity-oriented approach to harm reduction is a key dimension of EOHC, as illustrated in Fig. 2. Simultaneously, we recognized that whereas we previously had conceptualized ‘contextual tailoring’ as a key dimension, it is a process that applies to the interconnected dimensions of cultural safety, trauma- and violence-informed care and harm reduction.

Based on an understanding of the intersections among trauma, violence, and substance use, an equity-oriented approach to harm reduction draws attention to the ways the harms of substance use are increased by social determinants of health such as low income and inadequate housing, and social experiences such as abuse, trauma, grief and loss. Such an approach will also draw attention to how stigma and discrimination exacerbate the harms of substance use and impede access to health care.

The role of the health care sector in fostering equity requires more than well-intentioned calls for EOHC. The dimensions of such care must be described, strategies systematically identified, and actions taken to dramatically shift how care is provided if the health care sector is to have an impact on inequity. We are using our evolving articulation of the key dimensions of EOHC and associated strategies as a basis for interventions within primary care, and beyond. We believe this evolving approach has promise for public health and acute care sectors, and hope that others will contribute to its development and join efforts toward productive disruption.

## Abbreviations

EOHC: Equity-oriented health care
MOA: Medical office assistant
OIT: Organizational integration and tailoring

## Footnotes

1 We use the term ‘marginalized’ to refer to the social, economic and political conditions that contribute to health and health care inequities, and to the disproportionate effects of such inequities on particular segments of the population.

2 Structural violence refers to the systemic disadvantages that stem from the perpetuation of unjust structures, policies, and institutional practices, which shape everyday social patterns and contribute to poor health. Structural violence is recognized as a major determinant of the distribution and outcomes of social and health inequities [44].

3 Consistent with accepted terminology used in landmark international reports, the term ‘Indigenous peoples’ is used to refer to Indigenous communities, peoples and nations who have historical continuity with pre-colonial and/or pre-settler societies. In Canada, over 1.4 million people (4.3%) of the total population of ~ 37 million identify as Indigenous [45], including First Nations, Métis, and Inuit people. The term Aboriginal is also commonly used, and the colonial term ‘Indian’ is still used in federal government policy documents (e.g., The Indian Act) [46].

4 Harm reduction is a philosophy and a set of programs and services focused on preventing the harms of substance use, not reducing substance use per se. From a harm reduction perspective, substance use is viewed as a health issue [47].
